# MultiOmicsAgent: Guided Extreme Gradient-Boosted Decision Trees-Based Approaches for Biomarker-Candidate Discovery in Multiomics Data

**DOI:** 10.1021/acs.jproteome.4c01066

**Published:** 2025-05-26

**Authors:** Jens Settelmeier, Sandra Goetze, Julia Boshart, Jianbo Fu, Amanda Khoo, Sebastian N. Steiner, Martin Gesell, Jacqueline Hammer, Peter J. Schüffler, Diyora Salimova, Patrick G. A. Pedrioli, Bernd Wollscheid

**Affiliations:** † Institute of Translational Medicine at the Department of Health Sciences and Technology, 27219 ETH, Zurich 8093, Switzerland; ‡ Swiss Institute of Bioinformatics, Lausanne 1015, Switzerland; § ETH PHRT Swiss Multi-Omics Center (SMOC), Zurich 8093, Switzerland; ∥ Department of Biology, ETH, Zurich 8093, Switzerland; ⊥ Institute of Pathology, TUM School of Medicine and Health, 9184 Technical University of Munich, Munich 81675, Germany; # Department for Applied Mathematics, 9174 Albert-Ludwigs-University of Freiburg, Freiburg 79104, Germany

**Keywords:** multiomics, machine learning, extreme gradient-boosted decision trees, Python tool, biomarker discovery

## Abstract

MultiOmicsAgent (MOAgent) is an innovative, Python-based open-source tool for biomarker discovery, utilizing machine learning techniques, specifically extreme gradient-boosted decision trees, to process multiomics data. With its cross-platform compatibility, user-oriented graphical interface, and well-documented API, MOAgent not only meets the needs of both coding professionals and those new to machine learning but also addresses common data analysis challenges like normalization, data incompleteness, class imbalances and data leakage between disjoint data splits. MOAgent’s guided data analysis strategy opens up data-driven insights from digitized clinical biospecimen cohorts, making advanced data analysis accessible and reliable for a wide audience.

## Graphical abstract

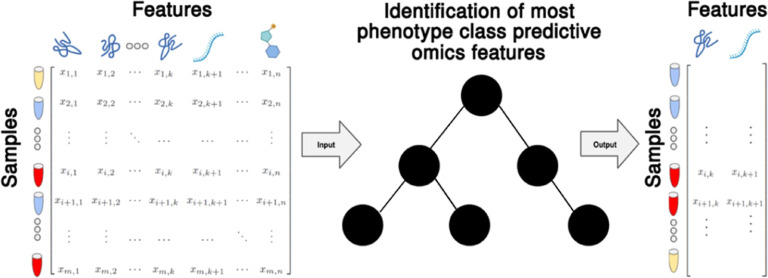

## 1 Introduction

The concept of a molecular digital twin, obtained through digitizing individual clinical biospecimens using multiomics strategies, offers new opportunities to gain diagnostic insights into human health.^1^ Next-generation sequencing-based transcriptomics, along with Mass Spectrometry (MS)-based proteomics and both MS and Nuclear Magnetic Resonance (NMR)-based metabolomics, are the de facto standard methodologies for the generation of quantitative data matrices representing the identity and abundances of thousands of transcripts, proteoforms, and metabolites across biological samples of interest.^2–9^ Identifying the subset of molecular entities that strongly associate with biological classes of interest from these quantitative matrices is an essential first step in understanding the underlying class-specific molecular biology and defining putative biomarker signatures.

Over the past decade, the advancements in the development of computational tools for multiomics analysis, particularly in enabling the detection of class-specific features, have been made by several researchers.^10–15^ Despite these advances, multimodal data analysis remains challenging. Analysts are required to efficiently manage incomplete data, complex combinations of multiple features, and false discovery rate (FDR) in the presence of limited replicates and large feature numbers. Indeed (multi)­omic studies are often burdened by the curse of dimensionality and high rates of missing values.^16^ This is often especially true in the context of analyzing data from clinical studies where sample cohorts of limited size and high heterogeneity can hinder the extraction of clinically relevant insights. Furthermore, interpreting the results from such analyses requires a strong understanding of the biological system under investigation. It is, therefore, desirable to make consistent multimodal data analysis accessible to a wide scientific audience, notwithstanding coding or machine learning expertise. This can be achieved via user-friendly interfaces and protection from common pitfalls and challenges such as class imbalances, overfitting, generalization, and information leakage.

Here, we present MultiOmicsAgent (MOAgent) as a solution, an easy-to-use, open-source, cross-platform compatible, graphical user interface-driven application for selecting phenotypic features from quantitative (multi)­omics data using machine learning. MOAgent can directly handle molecular expression matrices - including proteomics, metabolomics, transcriptomics, as well as combinations thereof. The MOAgent-guided data analysis strategy is compatible with incomplete matrices and limited replicate studies and was tested for sample sizes up to 10,000 and feature numbers up to 100,000.

## 2 Materials and Methods

### 2.1 Implementation and Features

The code for the MultiOmicsAgent (MOAgent) application comprises two Python packages that provide backend (https://github.com/Wollscheidlab/MOBiceps) and frontend (https://github.com/Wollscheidlab/MOAgent) functionalities. The former includes a number of functions centered around the selection and evaluation of phenotype-relevant features from quantitative omics data and exposes a well-defined API for programmatic control by a coding-competent audience. The latter provides an intuitive GUI and streamlines access to these functions for users who might lack coding or machine learning expertise. In addition to GitHub and pip distributions, MOAgent is also available as a downloadable virtual machine.

The core functionality of MOAgent can be accessed via the “RFE++” section of the GUI (Figure 1). From this panel, input files and analysis parameters can be specified before starting the workflow. The newly implemented main algorithm used in the selection of phenotypic features is based on an improved version of the recursive feature elimination approach by Guyon et al.^17^ we first implemented and we first implemented and used in Goetze et al.^18^ and Wildschut et al.^19^ hereby referred to as RFE++. The new algorithm is also a Monte Carlo-Simulation of tree based models, but we enhanced the compatibility for data with missing values (incompleteness), imbalanced class distributions, small data sets, multiclass and multiomic studies. The tree-based model can handle missing values without performing an imputation directly and makes sure that results are not biased by an imputation technique. Moreover, normalization techniques are usually not required for tree-based models, since they are feature scale-invariant, compared to other popular models like Logistic Regression, K-Nearest Neighbors, Support Vector Machines, and Neural Networks, etc. These properties of tree-based models make them a good choice for MOAgent to support multiomics data sets. Furthermore, the iterative and bootstrapping functionality of MOAgent improves the compatibility with small data sets and imbalanced class distributions. These effects, in combination with the provided cross-platform compatibility and graphical user interface, make our presented tool more applicable for a wide range of data sets without the need of adjusting the code.

**Figure 1:**
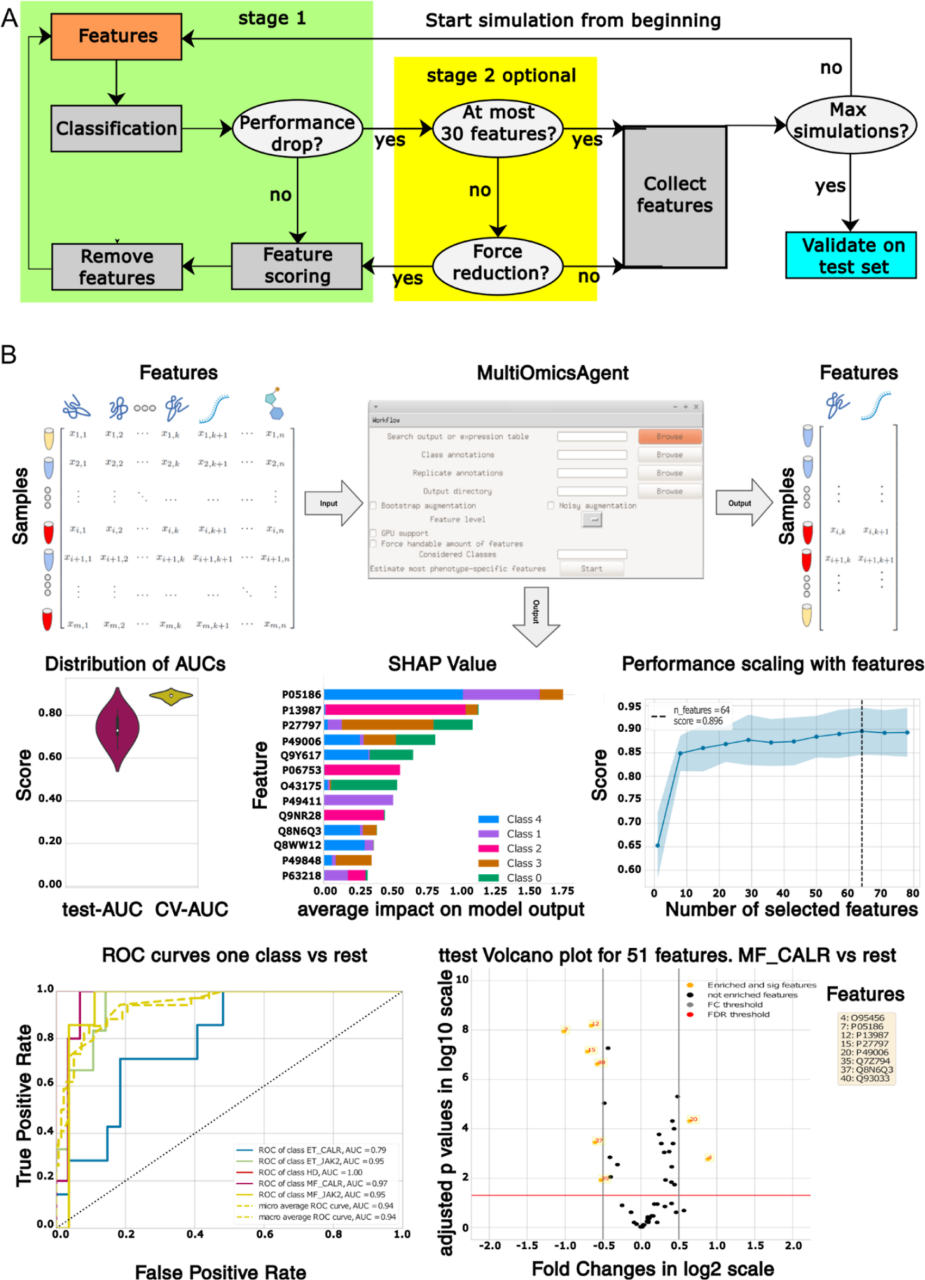
A, a flowchart illustrating how the RFE++ algorithm estimates the most phenotype-discriminating features iterating stages 1 and 2. B, the MOAgent graphical user interface (GUI) takes tabular data as input and generates an extensive output, including the most phenotype-discriminating features, model performances, and contribution evaluations of the features.

The input of MOAgent consists of:

- A CSV formatted quantitative (multi)­omic matrix with samples on the rows and features (peptide/protein/transcripts/metabolites) on the columns is required. The last column specifies the classes of the samples. Note that for proteomic studies, the output of a Spectronaut^20^ data analysis (exported to iq.rs report format) and Fragpipe (using a DIA_SpecLib_Quant workflow for data-independent acquisition (DIA) or LFQ-MBR workflow for data-dependent acquisition (DDA)) are also directly supported as input (see Figure S1 for an example). If Spectronaut or Fragpipe/DIA-NN search output is used as input for MOAgent, the user has to specify with the parameter ″Feature level″, if MOAgent should use peptide or protein feature level. In general, it is favorable to use an input with a symmetric distribution of values as it is often approximately achieved with log transforms in proteomics, metabolomics or transcriptomics expressions.
- A class annotation file specifying the relationship between samples and phenotypic classes. This file has to be formatted as a CSV and contains the two columns “files” (with file extension) and “class” (see Figure S2 for an example).
- An optional replicate annotation file specifies which samples are replicates of each other. This file has to be formatted as a CSV and contains the two columns “files” (with file extension) and “SampleID” (see Figure S3 for an example). A SampleID can correspond to a patient, for example, for whom we might have three replicates. When provided, this information will be used to avoid splitting related information across training, validation, and test sets, which is a very common and critical mistake.

The RFE++ algorithm is designed to find the most contributing features for phenotype classification. Since classes are often unbalanced, particular attention has been given to this aspect during the implementation of the selection procedure. For example, the area under the microaveraged receiver operating characteristic (ROC) curve representing the true positive rate over the false positive rate for several decision thresholds (invariance of decision threshold) and classes is used to take class imbalance into account and to prevent the procedure from indiscriminately assigning samples to the most abundant class. Additionally, class distribution is maintained when generating training, validation, and test splits to achieve similar distributions across all splits. Finally, to account for the small number of samples often found in biological data sets, we also implemented a bootstrapping procedure based on repeated stratified sampling from the data set while adding conditional Gaussian noise for each feature given the phenotype class. This allows for a proper stratified representation of all classes within each cross-validation fold used during the cross-validation training. For each sample, the bootstrapping procedure constructs two additional temporary artificial replicates within the train folds (preventing information leakage across folds due to bootstrapping, which is a common issue) for each cross-validation iteration to help stabilize the algorithm.

At its core, our selection algorithm (Figure 1A) has been implemented as a Monte Carlo-like sampling of recursive feature elimination procedures. More specifically, a randomly stratified sampled subset of the data is first set aside as a final test set. The remaining data is randomly stratified split into a training and simulation internal test sets. Subsequently, an eXtreme Gradient Boosting (XGBoost) trees classifier is trained and cross-validated on the training data using a stratified 5-fold cross-validation, and the model with the best performing features is returned. These features are ranked according to their classification contribution given by their information gain, the worst 10% are removed, and the training/validation step is repeated. In this first stage, the process of recursive feature elimination is repeated, considering the remaining features, including not yet removed features that were ties to previously removed features, until a significant loss in classification performance is observed, which is guaranteed by the design of the algorithm. The significant loss is defined as a decrease in cross-validated ROC AUC by 0.1 of the current iteration’s CV ROC AUC compared to the CV ROC AUC of the previous rfecv iteration performed in stage 1. For the evaluation of the classification performance depending on the number of features, a series of scalability plots are created within each simulation for each repetition to the specified output folder and named rfecv_iter_{j}_sim_{i}_cv_roc_auc_ovo_weighted.pdf, where {j} being the rfecv repetition index and {i} the simulation iteration index. The list of features still being considered prior to the loss is saved to an output file (i.e., optimal_features_{i}.csv {i} being the simulation iteration index of stage 1) and is available to the user in the specified output folder. In a second, optional stage, if this list contains more than 30 features, the feature elimination is forced to continue until a maximum of 30 features remains. The number 30 was empirically derived as a trade off for reducing all features to an handable amount of subfeatures for further downstream analysis and still achieving a very strong classification performance and thus a strong reliability for the differentiation ability of these features across investigated classes. Once again, the remaining features are saved to a second output file (i.e., stage_2_forced_reduced_features_with_best_performance­{i}.csv). While not necessarily optimal for classification purposes, this smaller list provides an optimal selection of features that can still be comfortably handled in biological follow-up studies. To measure the classification performance of those 30 features, a random grid hyperparameter optimized XGBoost classifier is trained in a 5-fold-cross validation mode on the train set and then validated on the internal test set. The classification performance of those features can be evaluated by consulting the corresponding receiver operating curves (i.e., grid2_train_ROC_AUC_relevant_features_{i}.pdf, grid2_test_ROC_AUC_relevant_features_{i}.pdf) and confusion matrices (i.e., confusion_matrix_Grid2_train_{i}.pdf, confusion_matrix_Grid2_test_{i}.pdf) of the training and test predictions. The entire feature selection procedure (stages 1 and 2) is repeated multiple times (three by default) after shuffling the columns of the input feature matrix and the features are collected in each Monte Carlo-like iteration.

Finally, the generalization performance is evaluated using the union of the features selected during each cycle on the set-aside test data set after a random grid hyperparameter optimized repeated 5-fold-cross validation training of a gradient boosted decision tree classifier (i.e., final_XGB_model.json) on the initial training and validation data set. Additionally, to provide a better overview of how the final phenotype-discriminative features (i.e., golden_features.csv) are related to the original features of the whole data set, we also provide a CSV file (i.e., significant_high_correlated_features_of_golden_features.csv) containing the Kendall correlations of those features as well as their corresponding p-values and Benjamini-Hochberg adjusted p-values of the correlations. The classification performance of the final phenotype-discriminative features is visualized in a ROC curve of the optimized XGBoost model (i.e., roc_auc_final_XGBclassifier_golden_features_test_performance.pdf).

In addition to the aforementioned output files, MOAgent also generates multiple graphical representations (Figure 1B) to help assess model performance and reliability. Information of console output is logged in “rfePlusPlusLog.txt”, including performance scores like precision, recall, f1-score and accuracy of cross-validation training and simulation internal tests for each iteration.

### 2.2 Setting up the MultiOmicsAgent (MOAgent) Virtual Machine

In line with our goal of lowering the adoption barrier of the presented workflow for users lacking coding experience, we decided to complement the pip-based package installation with a fully preconfigured virtual machine (VM) available for download as a zip-compressed file from Zenodo (https://zenodo.org/records/14446385). The VM seamlessly operates using the established open-source VirtualBox (version 7) software and has been tested on Intel-based macOS (>10.7), Ubuntu (versions 20.04 and 22.04), and Windows (versions 10 and 11) systems.

To use MOAgent via VM:

1. Install VirtualBox for your Operating System [https://www.virtualbox.org/].
2. Download and extract the VM file from Zenodo [https://zenodo.org/records/14446385].
3. Navigate to the extracted folder and double-click on the MOAgentVM.vdi file.

This will start a fully prepared Xubuntu Desktop - an open-source, lightweight operating system - featuring a MOAgent desktop shortcut alongside some demo files in the “Demo” folder that can be used for testing. The user name is moagent and default password 123.

To access data in MOAgent, we recommend to upload your data to a cloud storage of your choice (e.g., Google drive) and then download the data within the VM, since an Internet connection is by default available if your host computer is also connected to the Internet.

As an alternative you can configure the VM to either access a folder on the host machine (e.g., Windows), an external USB storage, or to use the popular preinstalled FTP client FileZilla within the VM. For more advanced users OpenSSH is also installed.

### 2.3 How to Use MOAgent, a Demo

After the user starts the VM and logs in with the default password 123, MOAgent can be started with the desktop shortcut “MOAgent Shortcut.” The application GUI of MultiOmicsAgent will start, as shown in Figure 1B. On the Xubuntu desktop, a folder named “Demo” is located. In this folder, all the input files of the Metabolomics (GN) case study are located.

1. Use the “Browse” button of the MOAgent GUI belonging to the field “Search output or expression table” to navigate to the Demo folder on the desktop, e.g., “/home/moagent/Desktop/Demo.” Open the “input” folder and select the “metabolite_expression_table.csv” file. The CSV file content is an expression matrix with the first column, “files” containing the file names, the last column, “class” containing the class annotations, and the columns between named according to the features associated with the expression values. An example is shown in Figure S1.
2. Use the “Browse” button of the MOAgent GUI belonging to the field “Class annotations” and navigate to the Demo folder on the desktop, e.g. “/home/moagent/Desktop/Demo”. Open the “input” folder and select the “class_annotations.csv” file. An example of the class_annotations.csv file is shown in Figure S2.
3. Use the “Browse” button of the MOAgent GUI belonging to the field “Output directory” and navigate to the Demo folder on the desktop, e.g. “/home/moagent/Desktop/Demo”. Select the “output” folder.
4. Check the checkbox corresponding to “Force handable amount of features” and press the “Start” button of the MOAgent GUI to run the analysis.

The results of the MOAgent analysis will be available in the folder “/home/moagent/Desktop/Demo/output/“. They will be similar to the reported results described in the GN cohort case study which can be also found in “/home/moagent/Desktop/MOAgent_supplementaries/case_studies_results/MetaboAnalyst_tut/“.

## 3 Results and Discussion

In this section, we apply MOAgent to multiomics, including transcriptomics, metabolomics and proteomics and multiclass data sets from previous published analyses to showcase MOAgent’s ability to find the most phenotype differentiating features in the data sets. Additionally, these data sets show typical challenges like class-imbalances, missing values, being measured across different facilities and match the usual magnitudes of sample sizes.

### 3.1 MultiOmicsAgent (MOAgent) in Methylmalonyl-CoA Mutase Deficiency Study: Confirmations and New Insights

#### 3.1.1 Transcriptome Case Study

We applied MOAgent to the transcriptome profile of the methylmalonyl-CoA mutase deficiency (MMA) cohort published by Forny et al.^21^ In this study, 122 patients showed no Methylmalonyl-CoA mutase (MUT) enzymatic activity and 64 showed normal activity. We log_2_ transformed, after adding element wise the constant 1, the values in the transcriptomics expression table of the original study to achieve a more symmetrical distribution. This is generally preferable for tree-based models such as the one used by MOAgent. MOAgent achieved a test microaveraged ROC AUC score of 76% (Figure 2A), suggesting that the list of selected transcripts was highly reliable. Among the top features in the SHAP summary plot, we found the MUT transcript (ENSG00000146085) (Figure 2B), also identified as a key disease-relevant transcript in the original study.

**Figure 2:**
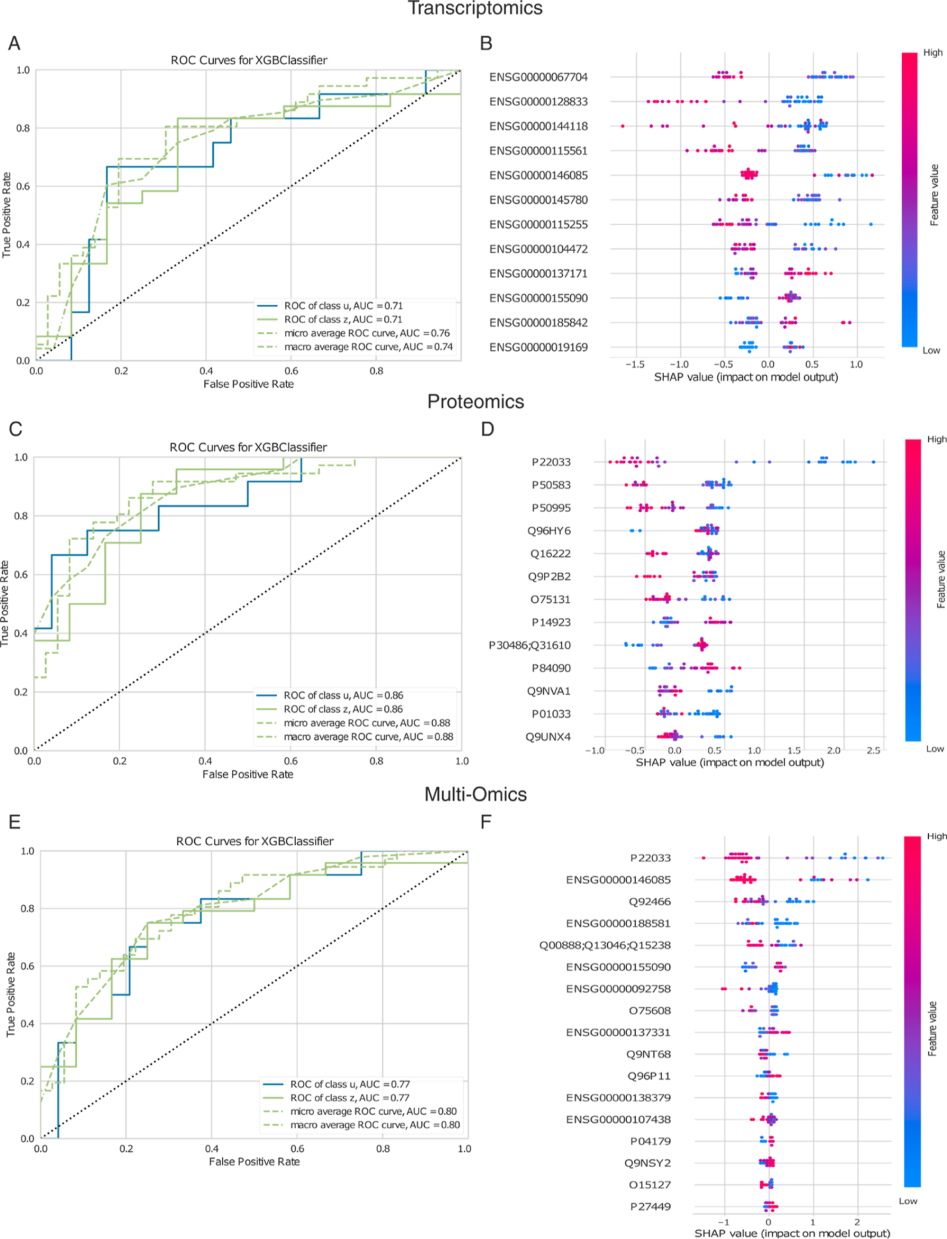
(A,C,E) Show respectively the ROC curves for the test set of the MUT active and inactive classification task based on transcript, protein and transcript combined with protein (multi omic) expressions. The corresponding micro average ROC AUC scores are 76%, 88% and 80%, respectively and indicate across all omics levels a high reliability of the extracted most classification-relevant features from the data set. (B,D,F) Show the corresponding SHAP summary plots and visualize the classification contribution of the extracted features, where features with a higher SHAP value deviation indicate a stronger class discriminatory power.

For this case study, no ″Feature level″, ″GPU support″, ″Bootstrap-″ or ″Noisy augmentation″ was selected. ″Considered Classes″ was not specified, which implies MOAgent will take the unique classes it found in the ″class annotation file″ that was provided. Only the ″Force handable amount of features″ was selected in the GUI.

#### 3.1.2 Protein Case Study

We further applied a similar workflow to the proteomics data published in the same study by Forny et al. The test micro averaged ROC AUC score for the classification was 88% (Figure 2C). Also, in this modality, the protein corresponding to the MUT gene (UniProt P22033) is selected and ranked first (Figure 2D).

For this case study, no ″Feature level″, ″GPU support″, ″Bootstrap-″ or ″Noisy augmentation″ was selected. ″Considered Classes″ was not specified, which implies MOAgent will take the unique classes it found in the ″class annotation file″ that was provided. Only the ″Force handable amount of features″ was selected in the GUI.

#### 3.1.3 Multi-Omics Case Study

Finally, we applied MOAgent on the concatenated transcriptomics and proteomics data from the MMA study. The test micro averaged ROC AUC score of the classification was 80% (Figure 2E). Once again, P22033 and ENSG00000146085 were among the top-selected features (Figure 2F).

For this case study, no ″Feature level″, ″GPU support″ was selected. ″Considered Classes″ was not specified, which implies MOAgent will take the unique classes it found in the ″class annotation file″ that was provided. Only the ″Force handable amount of features″, ″Bootstrap augmentation″, and ″Noisy augmentation″ were selected in the GUI, because of the increasing amount of features by using both transcriptomics and proteomics features.

### 3.2 MOAgent Easily Retrieves Known Biomarker-Candidates of a Complex Myeloproliferative Neoplasms (MPN) Cohort

We applied MOAgent on the myeloproliferative neoplasms (MPN) cohort published by Wildschut et al.^19^ This study includes multiple phenotypic classes of blood cancer disease. The two major forms of MPN (Myelofibrosis (MF) and essential thrombocythemia (ET)), are mainly driven by mutations in the CALR and the JAK2 genes. Additionally, blood from healthy donors (HD) with matching gender and age were used as a control class. The five phenotypic classes were, therefore, designated HD, ET CALR, ET JAK2, MF CALR, and MF JAK2, respectively.

In the original publication, we identified 21 proteins that could be used to achieve high classification accuracy between the five phenotypes. Here, we first applied the DIA_SpecLib_Quant workflow of Fragpipe—a Java GUI that provides a comprehensive suite of computational tools for analyzing mass spectrometry-based proteomics data—on the raw MS files from the study (ProteomeXchange data set PXD036075) to generate a file capturing the protein expressions (i.e., diann.tsv). In combination with the patient- and class-annotation CSV files and the ″Bootstrap augmentation″ and ″Noisy augmentation″ functions to derive, with one click, a list of phenotype-specific proteins via MOAgent (see Table S1). Three (i.e., NEK5, STYX, and ZNF735) of the 21 proteins selected in the original study were not identified by Fragpipe. The output of MOAgent contained 15 highly correlated or identical proteins for the remaining 18. Notably, while the original analysis was performed with Spectronaut—a commercial software package developed for analyzing DIA proteomics experiments—and included an ad-hoc programmed batch regression, missing value imputation, and feature selection pipeline, our reanalysis entirely relied on open-source software. It completely bypassed the need to write code for the analysis. It should also be mentioned that in the original publication, we used an earlier implementation of the RFE that optimized Extreme Randomized Decision Trees (Extra Trees) toward the macro averaged F1 score (i.e., the class averaged harmonic mean between precision and recall at one specific decision threshold), whereas MOAgent uses an XGBoost classifier and optimizes the model regarding micro averaged ROC AUC, considering the trade-off of true positives over false positives for a range of decision thresholds. Therefore, the newer implementation provides a classification threshold-independent evaluation and, thus a more meaningful assessment of the selected features’ discriminatory power. All in all, 83% of the class-relevant proteins detected in the original study were identified or highly Kendall correlated alternatives (after multi testing correction with Benjamini-Hochberg correction) of those by MOAgent with a test micro averaged ROC AUC score of 94% (Figure 3A,B) as highly class-relevant without needing to write a single line of code.

**Figure 3:**
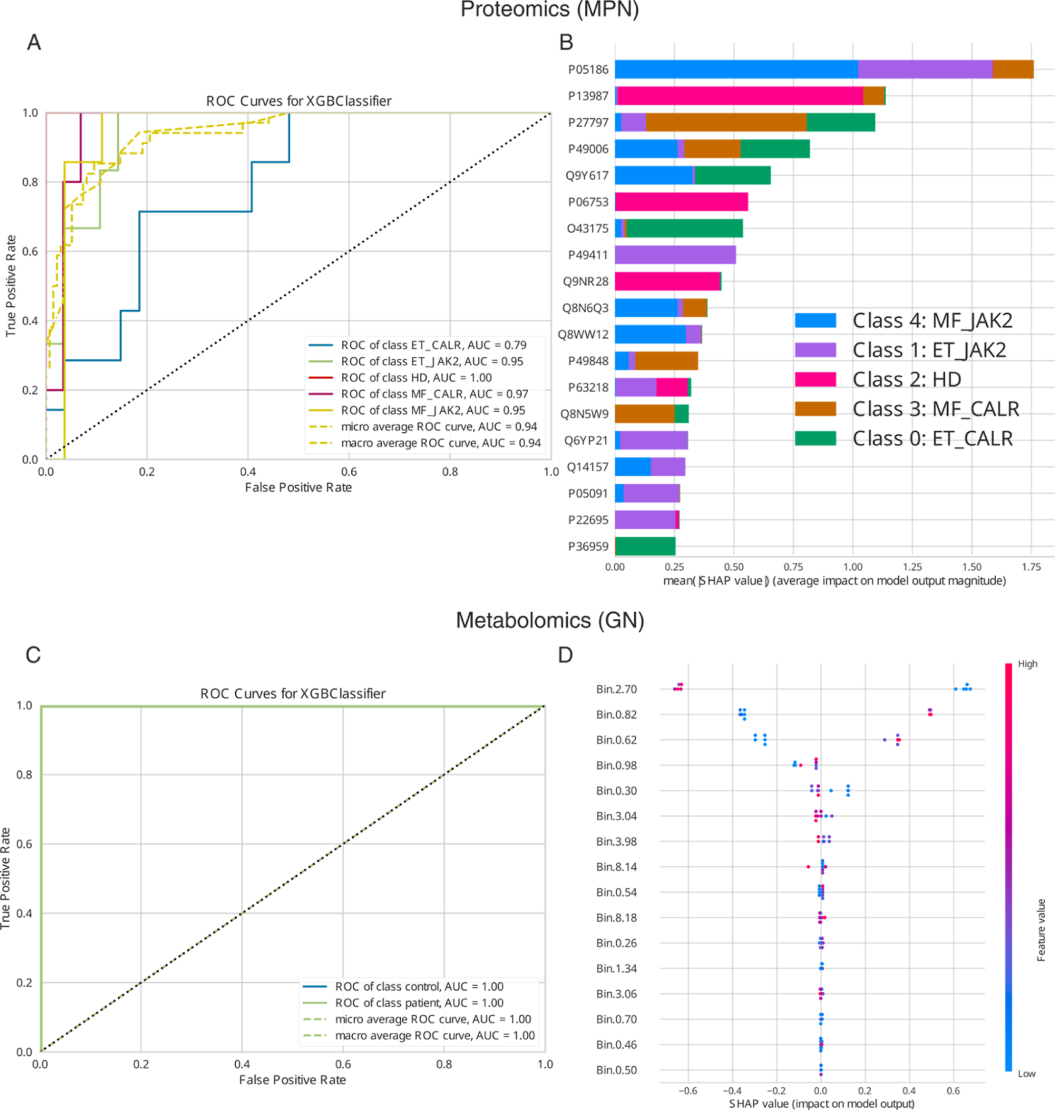
(A) Shows the ROC curves for the test set of the myeloproliferative neoplasms phenotypic classification task based on protein expressions. The corresponding micro average ROC AUC scores 94% and indicates a high reliability of the extracted most phenotype classification-relevant features from the data set. (B) Shows the corresponding SHAP summary plot and visualizes the classification contribution of the extracted features of the MPN classification task. (C) Shows the ROC curves for the test set of the Glomerulonephritis (GN) classification task based on metabolite expressions. The corresponding micro average ROC curve AUC scores 100% and indicates a high reliability of the extracted most classification-relevant features from the data set. (D) Shows the corresponding SHAP summary plot and visualizes the classification contribution of the extracted features of the GN classification task.

For this case study, the ″Feature level″ was chosen to be ″protein″. ″GPU support″ was not selected. ″Considered Classes″ was not specified, which implies MOAgent will take the unique classes (HD, ET CALR, ET JAK2, MF CALR, and MF JAK2) it found in the provided ″class annotation files″. The parameter ″Force handable amount of features″ was selected. Further, the parameters ″Bootstrap augmentation″ and ″Noisy augmentation″ were selected in the GUI, because of the small sample sizes per class in this case study.

### 3.3 MOAgent Perfectly Classified Glomerulonephritis Using NMR Data, Pinpointing Citrate as Crucial, without User Coding

We applied MOAgent to a metabolite expression table generated by Nuclear Magnetic Resonance (NMR) for the classification problem that is provided by MetaboAnalyst.^11^ The classification tutorial from this Web site contains 25 healthy controls and 25 Glomerulonephritis (GN) affected patients, a subset from the study of Psihogios et al.^22^ The super bin 2.70 ppm corresponding to citrate is known from the study as a key player in the disease and was identified as the top feature in the MOAgent SHAP summary plot (Figure 3C). For this study, the test micro averaged ROC AUC score of the classification was 100% (Figure 3D). Once again, these results were achieved without writing a single line of code by the user.

For this case study, no ″Feature level″, ″GPU support″ was selected. ″Considered Classes″ was not specified, which implies MOAgent will take the unique classes it found in the ″class annotation file″ that was provided. Only the ″Force handable amount of features″, ″Bootstrap augmentation″, and ″Noisy augmentation″ were selected in the GUI, because of the small sample sizes for each class in this case study.

### 3.4 MOAgent Validated Cell Clonality in Multiple Myeloma with High Accuracy, Highlighting Immunoglobulin Light Chains as Key Features

We applied MOAgent to validate the clonality of CD138-sorted cells from a multiple myeloma patient cohort published by Kropivsek, K. et al.^23^ The quantitative CD138 protein data matrix contained a total of 74 samples and two phenotypic classes (i.e., 48 samples with Kappa light-chain and 26 with Lambda light-chain). MOAgent achieved test micro averaged ROC AUC scores of 91% on the protein level (Figure 4A) and 95% on the peptide level (Figure 4C). As expected, at the protein level in the SHAP summary plot, we find features of the immunoglobulin Kappa and Lambda light chain as the two most significant hits (Figure 4B). Since we had replicates from patients, we used a ″patient annotation file″. The classes were derived from the unique classes found in the ″Class annotations file″. We did not use ″GPU support″ and no ″Bootstrap- or Noisy augmentation″. As ″Feature level″ we selected ″protein″. We also selected the ″Force handable amount of features″ parameter.

**Figure 4:**
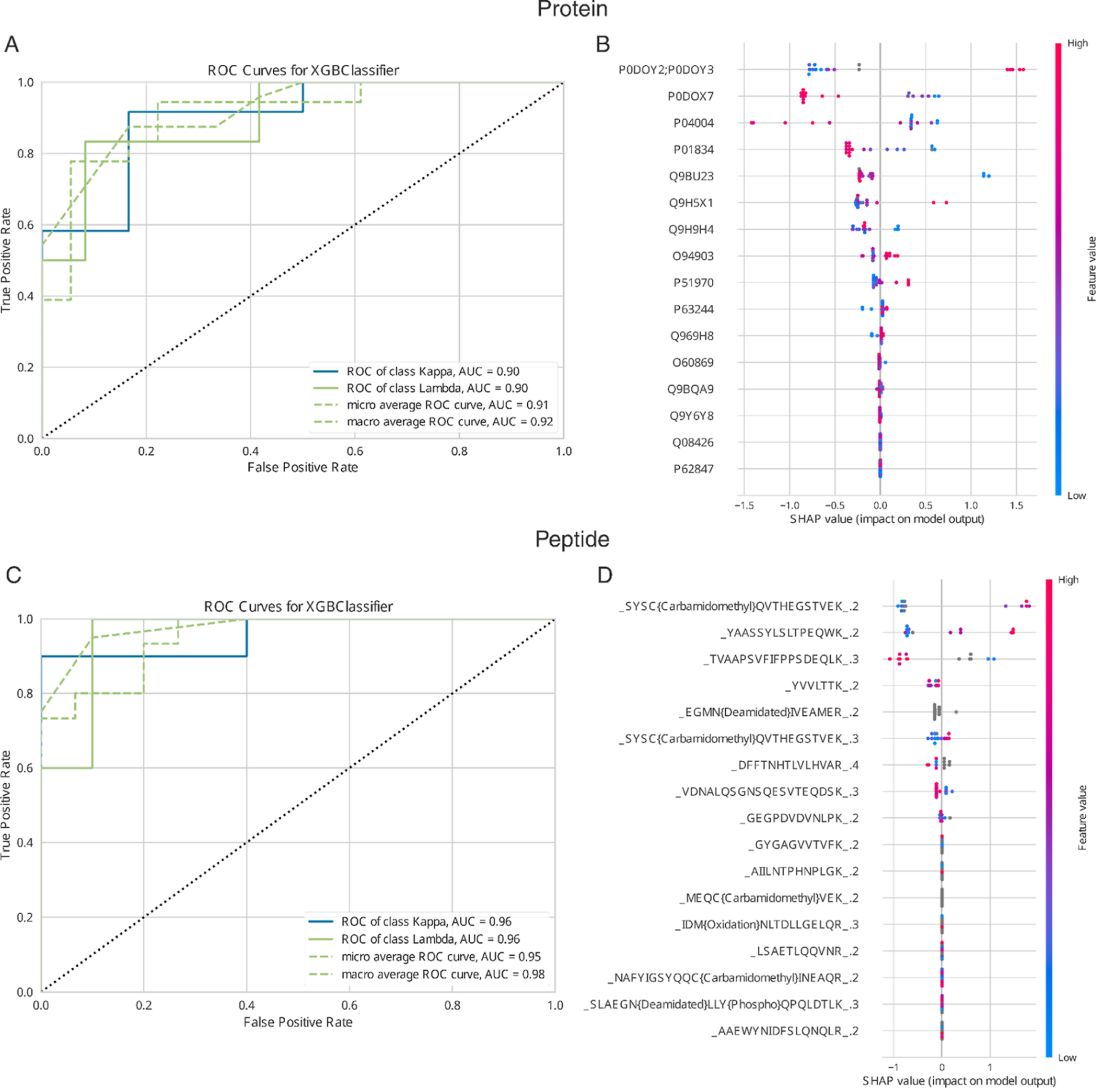
(A,C) Show the ROC curves for the test set of the clonality in multiple myeloma (Kappa–Lambda) classification task based on protein and peptide expressions, respectively. The corresponding micro average ROC AUC scores are 91% and 95%, indicating a high reliability of the extracted most classification-relevant features from the data set. (B,D) Show the corresponding SHAP summary plots and visualize the classification contribution of the extracted features of the Kappa–Lambda classification task.

Similarly at the peptide level, we find peptides from the immunoglobulin Kappa and Lambda among the top-ranking features for classification (Figure 4D). Once again, these results were achieved without a single line of code. The parameter setting for the peptide level was the same as for the protein level, except of having selected ″peptide″ for the ″Feature level″ parameter and ″Bootstrap- and Noisy augmentation″. The augmentations were chosen due to the drastic increase of feature size on the peptide level compared to protein level.

### 3.5 New Insights from the Case Studies Analysis Using MultiOmicsAgent (MOAgent)

The integration of MOAgent within the realms of protein and transcriptomic analysis, or broadly across multiple omic levels, has unveiled novel perspectives on the comparative contributions of various omic features to phenotypic manifestations. In studies focusing on proteins and transcriptomes, the AUC test scores revealed a nuanced balance between true positive and false positive allocations of samples to phenotypic categories, with protein expressions yielding a superior trade-off at 88%, compared to 76% in the transcriptomic analyses of the MMA cohort. In the Kappa-Lambda case study an improvement of 4% on the peptide level compared to the protein level can be observed supporting the focus on peptide level for downstream proteomics analysis as already discussed in Plubell et al.^24^

MOAgent allows not only multiomics studies, it enables also multiclass studies in the same framework as showcased in the MPN cohort example. Figure 3A reveals the ET CALR class is more challenging to classify than any other of the classes, which can be concluded by the class-specific ROC curves MOAgent provides.

When examining multiomic studies, MOAgent facilitates a unique confrontation among features from disparate omic strata, highlighting the most discriminative attributes for class separation. Notably, the MMUT corresponding protein in the MMA study and its transcript emerge as leading indicators, as determined by SHAP values. This cross-omic rivalry uncovers biologically significant features that might elude detection within isolated omic investigations. For instance, a transcript deemed critical for classification may not translate into a discernible protein expression within phenotypic variations, despite its association with a protein complex that includes a more influential protein member. Such discrepancies might stem from post-translational modifications or insufficient expression variances across phenotypic classes, given the sample size. Therefore, MOAgent’s SHAP-informed feature ranking presents an innovative lens for dissecting phenotypic nuances on a systemic biological scale supporting advances toward biological digital twins through multiomic integration.

Further insights from the MMA cohort underscore the strategic value under certain circumstances of prioritizing protein expressions, which achieved an AUC score of 88%, surpassing the 80% score from a combined protein-transcript approach.

This finding suggests that, depending on the objectives of a study, significant resources could be conserved by focusing on a single omic dimension, thereby streamlining the analytical process without necessarily compromising the depth of phenotypic understanding. Nevertheless, the incremental improvement observed in the MMA study, where the integration of protein and transcript expressions enhanced the true positive versus false positive ratio by 4%, underscores the potential of multiomic approaches to refine phenotypic classifications beyond the capabilities of single omic analyses.

## 4 Comparisons with Other Models

### 4.1 Data Generation

We employed the *make_moons­()* function from the sklearn library to generate a synthetic data set, emulating two interleaving half circles, which inherently lack linear separability, akin to two crescent moons. The data set comprises 80 points, further expanded by the addition of 5000 noninformative features. These additional dimensions were populated using random values drawn from a Gaussian distribution with a mean of zero and a variance of one, culminating in a real-valued data matrix with 80 samples across 5002 features. Critically, only two of these features are class-informative.

### 4.2 Rationale for Data Set Choice

The chosen data set simulates the complex, nonlinear separability prevalent in intricate omics data sets of a clinical cohort stratified across two classes with 40 samples for each class, where numerous features might not directly contribute to the phenotype classes under study. This data set’s absence of missing values eliminates the need for imputation, facilitating a more streamlined comparison with other models.

### 4.3 Data Splitting

We conducted a random stratified split of the data, designating 30% of the samples for testing following the model training phase, utilizing the robust sklearn framework.

### 4.4 Model Evaluation

For comparative analysis, we integrated Recursive Feature Elimination with Cross-Validation (RFECV) using logistic regression (LR), and an XGBoost classifier (XGB) from the XGBoost Python package. We also tested standalone LR, and XGB. Moreover, we included our RFE++ workflow from the MOBiceps Python package. Since RFE++ obviates the need for a predetermined amount of feature reduction, unlike usual Recursive Feature Elimination (RFE) methods, we have chosen the RFECV function of sklearn, which also obviates the need for a predetermined amount of feature reduction.

### 4.5 Performance Metrics

In our evaluations, the RFE++ implementation from MOBiceps, utilized within the MOAgent user interface, notably achieved a microaveraged ROC AUC of 0.87, as shown in Figure 5. Comparative performance for RFECV implementations showed the XGB model at a 0.45 microaveraged ROC AUC, and the RFECV with the Logistic Regression (LR) at 0.67. The standalone XGB and LR achieved 0.69 and 0.66, respectively. Overall, MOAgent outperforms the competing models by at least 26% in relative microaveraged ROC performance.

**Figure 5:**
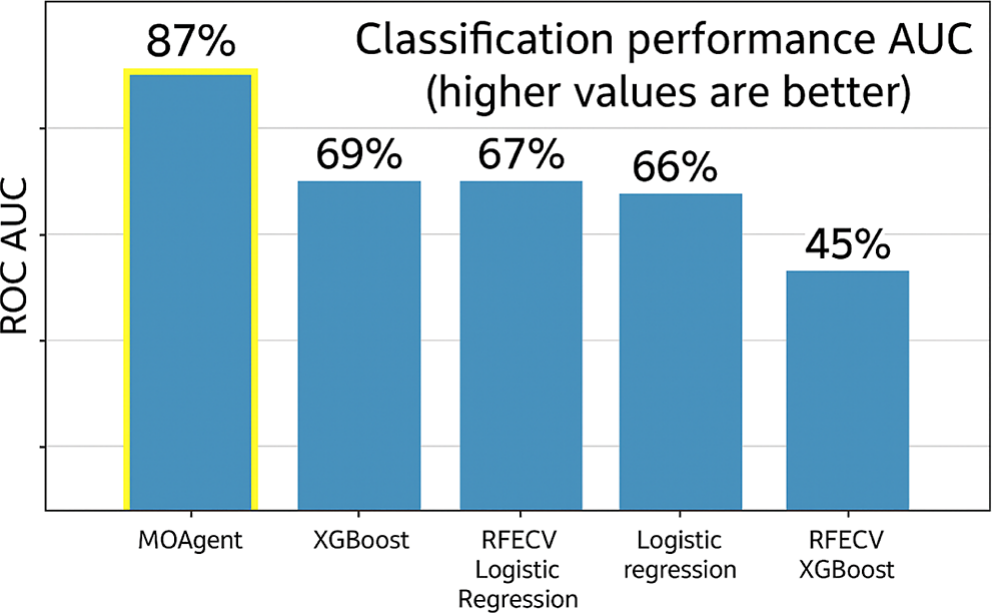
Microaveraged ROC AUCs of different models competing with MOAgent (yellow highlighted) in the synthetic moons data set.

## 5 Time and Memory Consumption

In this section we want to provide an overview of the time and memory consumption with increasing sample sizes and feature sizes to allow a user to make a rough estimation for their own data sets they want to process with MOAgent. We used an Ubuntu 20.04 workstation for the following experiments with an AMD Ryzen Threadripper Pro 3955WX CPU.

We employed the *make_moons­()* function from the sklearn library to generate a synthetic data set, emulating two interleaving half circles, which inherently lack linear separability, akin to two crescent moons. The data set is expanded by the addition of 198 noninformative features. These additional dimensions were populated using random values drawn from a Gaussian distribution with a mean of zero and a variance of one, culminating in a real-valued data matrix with 200 features. Critically, only two of these features are class-informative.

We investigated the time and memory consumption of the RFE++ function from the MOBiceps package, which is utilized in MOAgent with an increasing amount of samples. The results are shown in Figure 6A,B.

**Figure 6:**
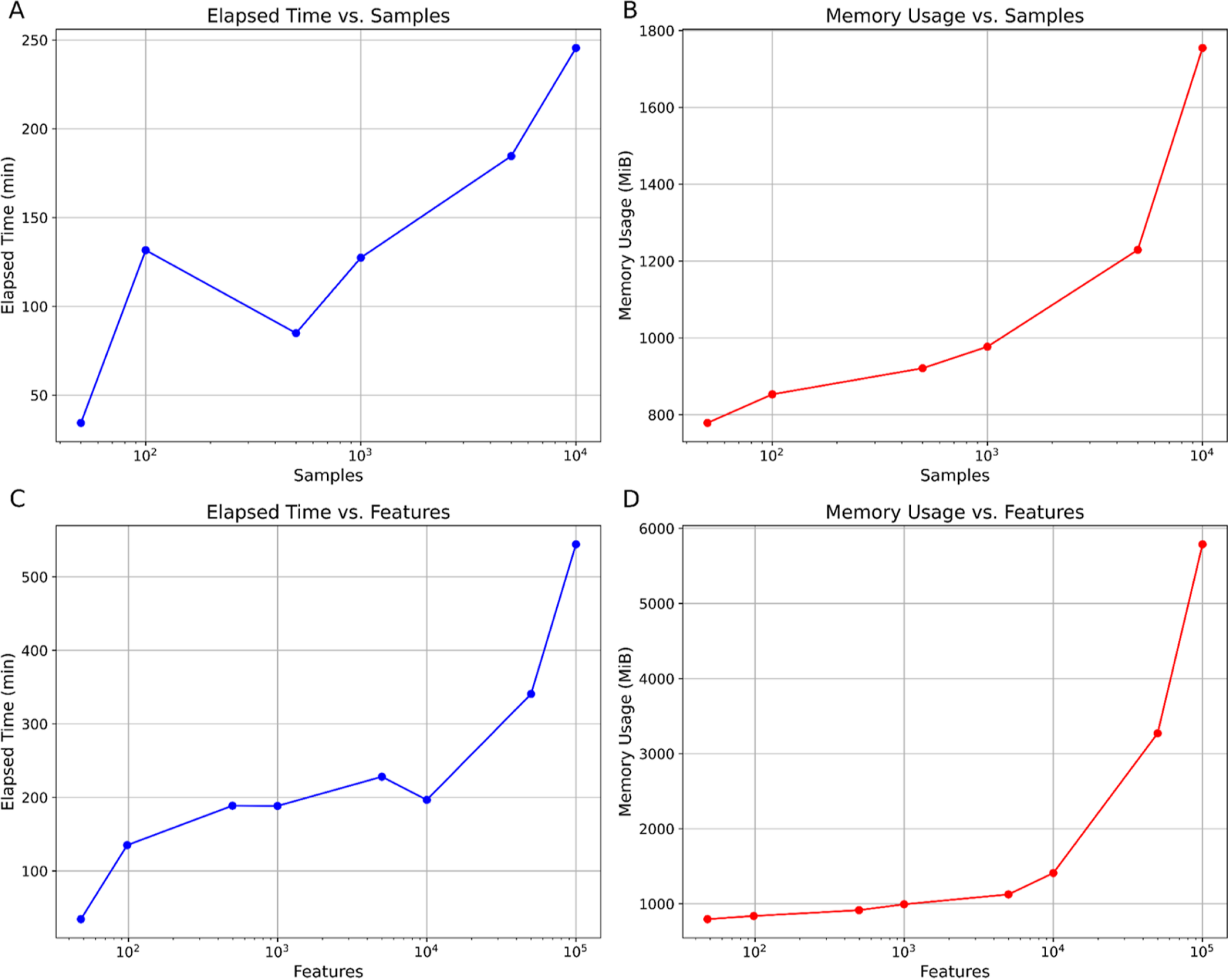
(A) Shows the wall clock run time of the RFE++ with an increasing number of samples. (B) Shows the memory consumption of the RFE++ with an increasing number of samples. (C) Shows the wall clock run time of the RFE++ with an increasing number of features. (D) Shows the memory consumption of the RFE++ with an increasing number of features.

We also investigated time and memory consumption while fixing the number of samples to 200 and increasing the number of noninformative features. The results are shown in Figure 6C,D.

## 6 Expectations for Real-World Applications

From the results,, we can draw the conclusion that MOAgent has exponential time and memory consumption with an increasing number of samples and features. Especially, requirements for sample and feature sizes over the order of 10^4^ are super exponential. However, for smaller sample sizes, which usually do not exceed 10^2^, the search for most predictive features takes roughly 2 h, if only a very small subset of features is informative. In a real-world example, we would expect more than two features to contribute to the investigated class, and thus can expect a runtime of less than 2 h using a typical workstation from 2021. If a user wants to process more than 10,000 samples with MOAgent, we recommend using MOAgent’s GPU support to accelerate the analysis.

For feature sizes in the order of up to 10^4^, which are often encountered on the peptide feature level or in multiomics studies, the memory and time consumption in a real world example is also expected to be less than 2 h, assumed the user works with an average workstation from 2021 and more than two features are contributing to the investigated class.

### 6.1 Automatic provided Data and Result Visualizations by MOAgent

#### 6.1.1 Feature Analysis and Visualization

(Figures S4 and S5) MOAgent generates both UMAP (Uniform Manifold Approximation and Projection) (see Figure S4) and PCA (Principal Component Analysis) (see Figure S5) plots, enabling a conclusion toward nonlinear and linear separability of data points regarding phenotypic classes, respectively. Conclusions considering similarities between different clusters should be taken from PCA plots, since the orthogonal mapping and projection onto the most variance contributing components (principle components) are more Euclidean distance preserving than the two UMAP components.

These plots are created considering the entire spectrum of features as well as focusing exclusively on the most phenotype-discriminating features and allows a qualitative investigation of class disentangling after the feature reduction. For successful feature selection, we anticipate the PCA and UMAP plots, when focused on phenotype-discriminating features, to exhibit less mixing and more structured clustering aligned with the phenotype classes of the data set.

#### 6.1.2 Performance and Reliability Evaluation

(Figure 7) To assess the classification performance and its generalization ability, MOAgent creates violin plots incorporating box plots. These plots visualize the distribution, quantiles, and medians of ROC (Receiver Operating Characteristic) and AUC (Area Under the Curve) scores for both cross-validation and test sets throughout the feature selection process (stages 1 and 2). Additionally, test scores for accuracy (ACC), macro averaged F1 score (MAVG_F1), which corresponds to averaging the unweighted mean per label, and weighted average F1 score (WAVG_F1), which corresponds to averaging the support-weighted mean per label, are visualized. Note that the decision thresholds of the classifier are optimized on the cross-validation set for maximum sensitivity (true positive rate) while minimizing the false positive rate. This decision threshold represents the best trade-off between correctly identifying positive cases while avoiding false positives. It corresponds to the point on the ROC curve that has the shortest distance to the top left corner (0,1) in the Cartesian coordinate system. However, we strongly recommend to focus on cross-validation and test AUC distributions to evaluate the features’ phenotype prediction capabilities.

**Figure 7:**
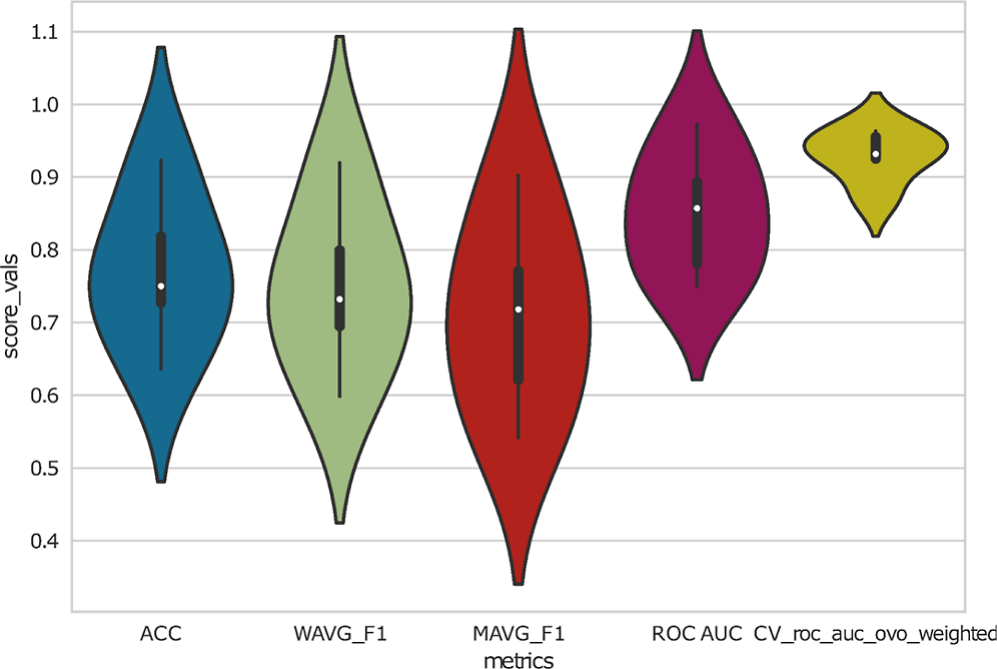
In each Monte Carlo-like simulation, a random grid hyperparameter optimized XGBoost classifier (named Grid2) is trained in a 5-fold cross-validation mode on a train set and additionally tested on a separate test set. This Figure from the Kappa-Lambda case study is shown as an example and visualizes the evaluation scores’ distributions in violin plots and box plots.

#### 6.1.3 ROC Curve Analysis

(Figure 8A,B) ROC curves are provided, illustrating the true positive rate against the false positive rate at various decision thresholds. This analysis is conducted for the XGBoost classifier in the final model and for training (Figure 8A) and test set (Figure 8B). The area under these curves serves as an indicator of model performance and should be preferred over confusion matrices to evaluate the predictive power of selected features. The bigger the area under the ROC curve, the better the model performs and the more reliable the selected features are.

**Figure 8:**
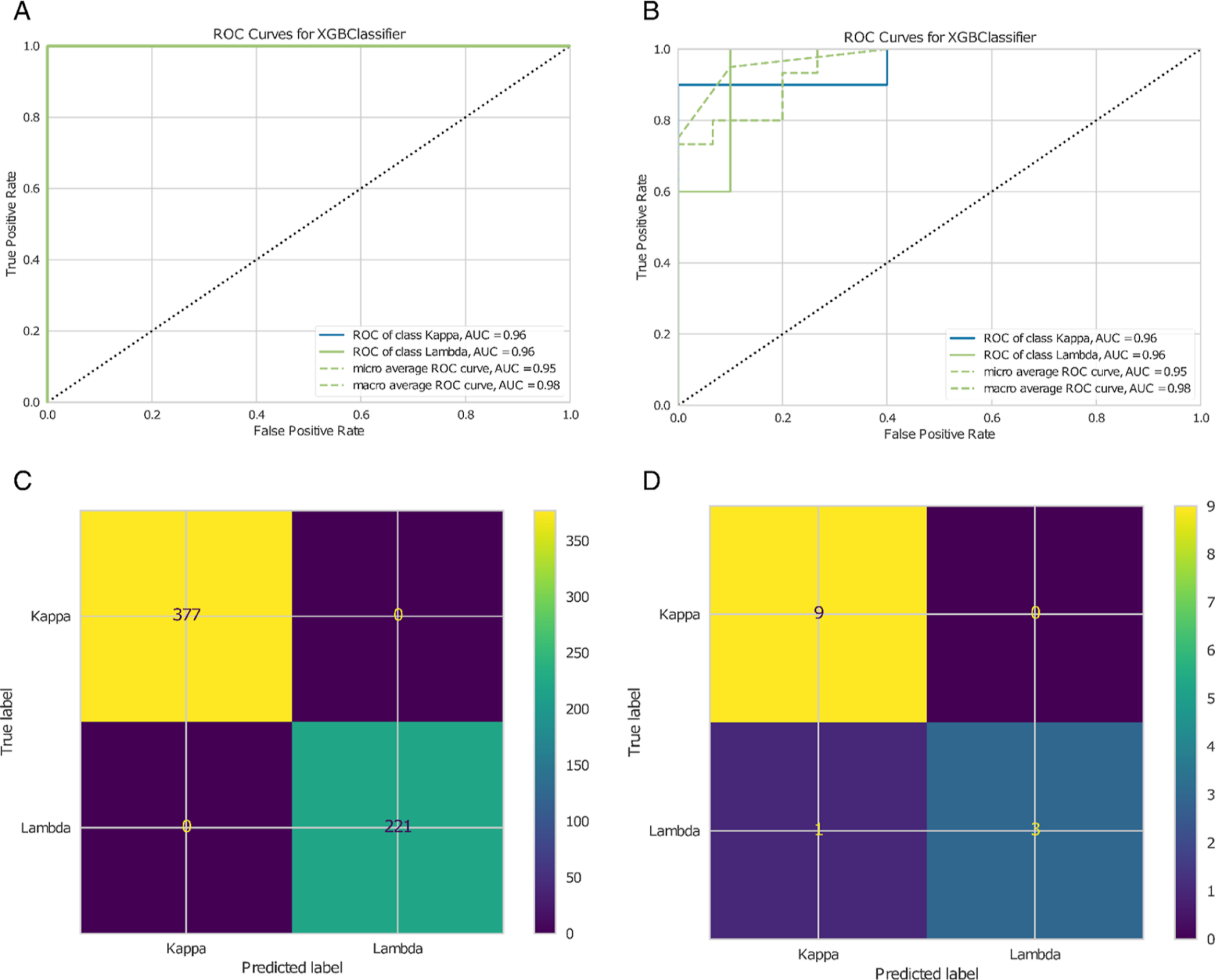
(A,B) Show example ROC curve for training and test predictions of the Kappa–Lambda peptide case study, respectively. (C,D) Show example figures for the train and test confusion matrices of the Kappa–Lambda peptide case study.

The roc_auc_final_XGBclassifier_golden_features_test_performance.pdf is the first of two key visual outputs a user should pay special attention to. Together with xgb_final_features_shap_test_performance.pdf (see “feature trustability visualization”), it illustrates the reliability of the selected features.

#### 6.1.4 Confusion Matrix and Further Statistics

(Figure 8C,D) Confusion matrices are generated in each simulation for train (Figure 8C) and test (Figure 8D) predictions of the hyperparameter tuned XGBmodel, allowing for the computation of additional statistical measures besides the ones provided, like accuracy, F1 score, sensitivity, and specificity, which are pertinent to the specific application. The better the classifier performs, the closer this matrix is to a diagonal matrix (all off-diagonal entries tend to be zero). The provided confusion matrices correspond to the final model using the decision threshold for which the train and test ROC curves respectively were closest to the top left corner (0,1) in the Cartesian system representing the true positive rate over the false positive rate.

#### 6.1.5 Feature Trustability Visualization

(Figure S6) Visualizations for direct measurement of the selected features’ reliability are provided through classical volcano plots (see Supporting Information Figure S6A) (after a data-dependent minimum value imputation for missing values) using two-sided Mann–Whitney U tests for each feature and SHAP (SHapley Additive exPlanations) (see Supporting Information Figure S6B) value plots. The volcano plots are computed for all features and specifically for selected features, with the latter being less stringent toward multitesting correction. Notably, both utilize Benjamini-Hochberg-adjusted p-values. In a multiclass phenotype classification scenario, multiple volcano plots (e.g., five for a five phenotype-class problem) are generated, each comparing one phenotypic class against the rest. If a user specifies only two classes in the GUI to investigate, then rest corresponds to a single comparison class. The SHAP value plot visualizes how much a feature contributed to the classification task, with more relevant features having bigger absolute values. The file xgb_final_features_shap_test_performance.pdf is the second of the two key visual outputs and shows the classification importance of the features in golden_features.csv.

#### 6.1.6 Correlation Analysis and Feature Distribution

(Figures 9 and S7 and S8) A hierarchical clustering with heatmap across the selected features is provided (see Figure 9). Further, the Kendall correlations of selected features are visualized in a heatmap (see Figure S7). The Kendall correlation is chosen, because it does not assume a specific feature distribution as a Pearson correlation does for example. Additionally, nonlinear relationships can be reliably captured. Kendall is also suitable for smaller data sets and is less sensitive to ties or very close feature values. MOAgent additionally provides boxplots (see Supporting Information Figure S8) that visualize the distribution of feature expression values across different classes.

**Figure 9:**
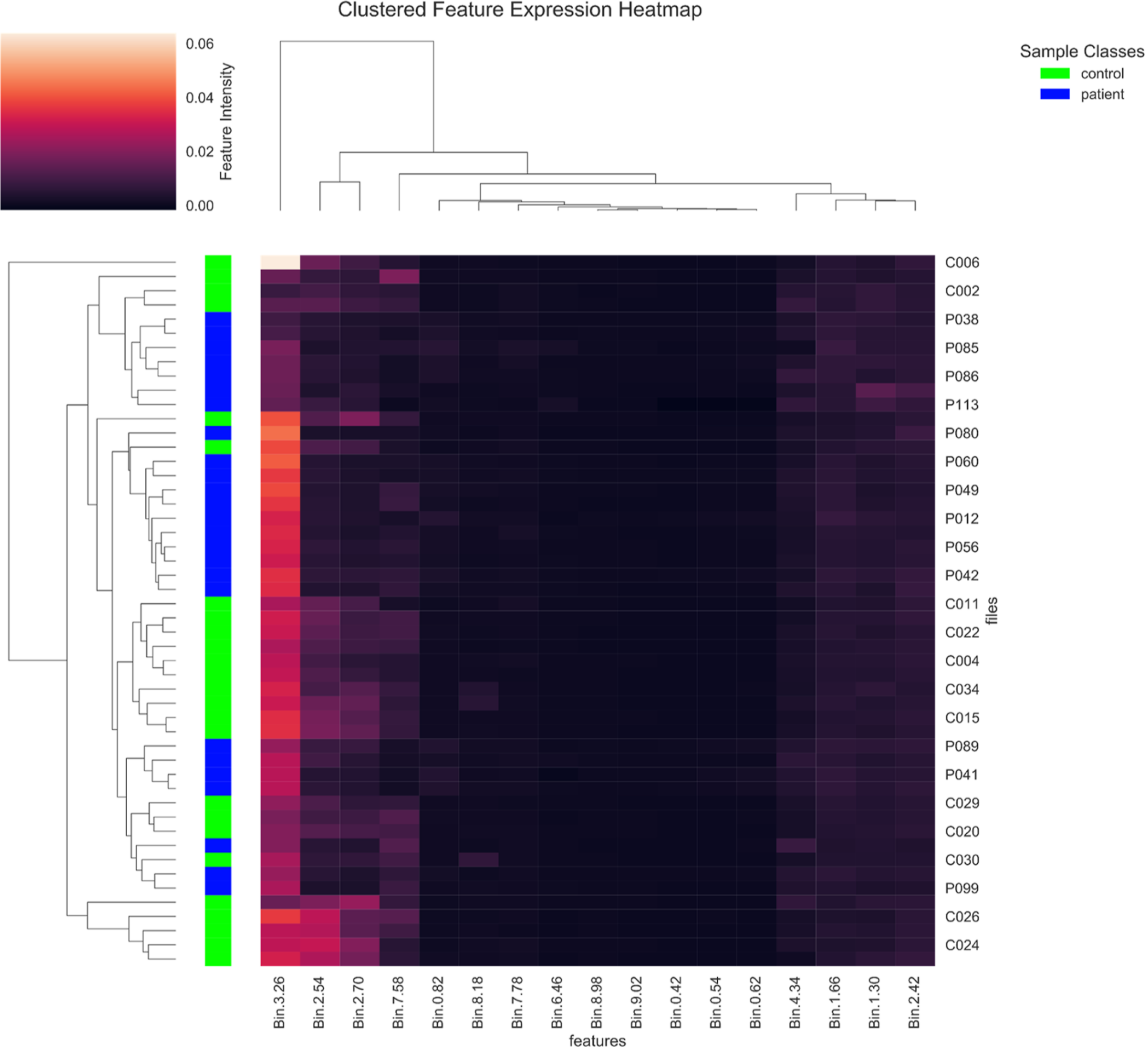
Selected feature expression heatmap from the GN case study.

#### 6.1.7 Feature Classification Performance Scalability

(Figure 10) In stage 1, an iteration of recursive feature eliminations is applied, and in each simulation for each recursive feature elimination repetition, a scalability plot is generated. One scalability plot shows the mean ROC AUC classification performance with standard deviation over the number of features.

**Figure 10:**
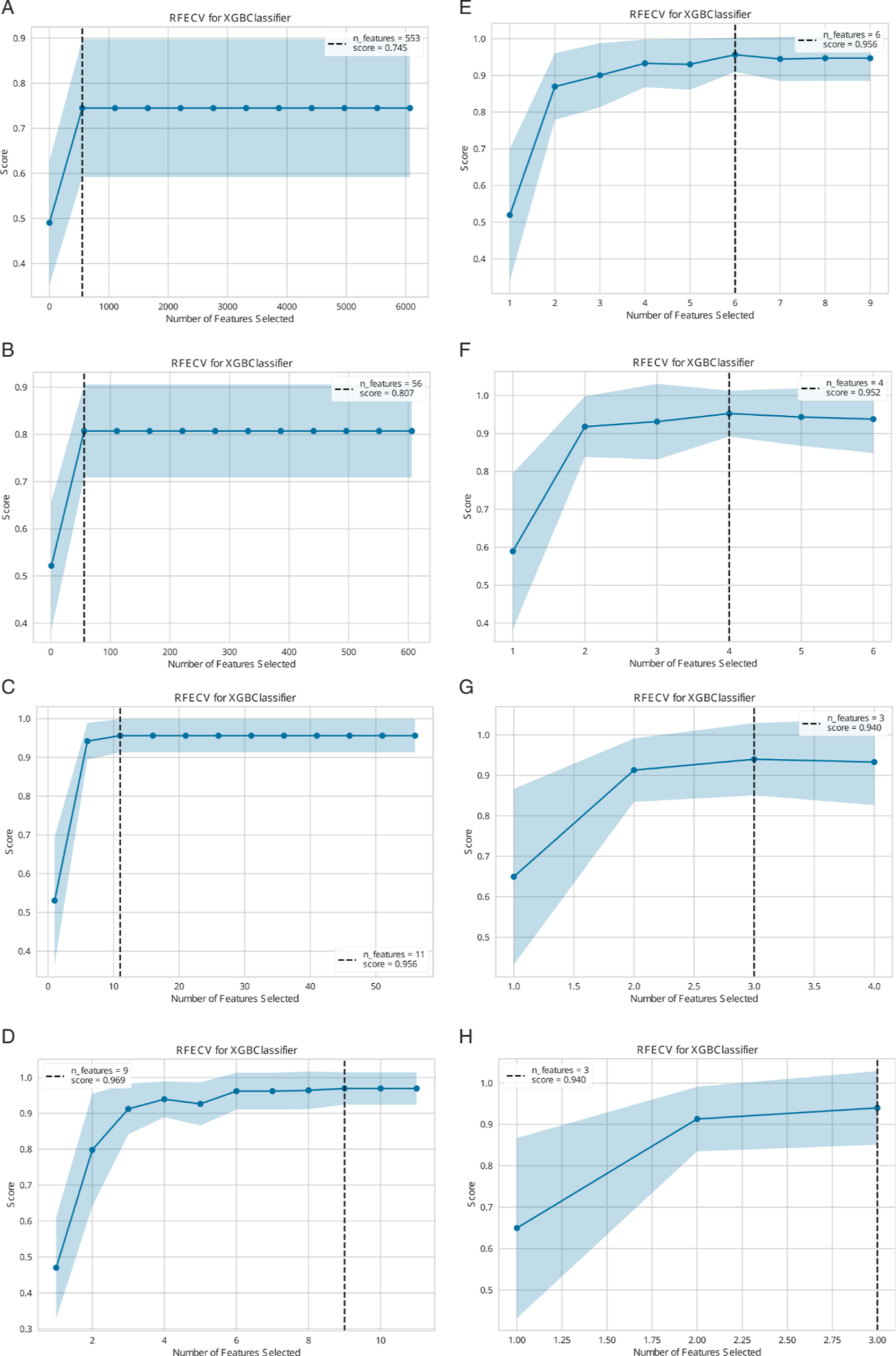
Scalability plots for the Kappa-Lambda case study on protein level. The iterations are sorted from top to down and left to right (A–H), indicated by the reducing maximum number of considered features from plot to plot.

## 7 Conclusion

While it has never been easier to identify and quantify thousands of molecular entities such as transcripts, proteins, and metabolites from biological samples, the extraction of biological significance from these multimodal data sets remains challenging and mostly confined to the realm of expert data scientists. Among the challenges that need to be addressed are the necessity to handle missing values, consider complex combinations of multiple molecular makeups (biomarker signatures), and efficiently control the false discovery rate in the presence of limited replicates and large feature numbers. Machine learning (ML) offers promising approaches to tackle this task if (as a rule of thumb) at least 20 data points per class in the data set are available. However, the accessibility of these methodologies is often restricted by high requirements in coding skills and ML domain knowledge. MultiOmicsAgent (MOAgent) addresses these concerns by providing access to an advanced, ML-based, multiomic biomarker candidate discovery pipeline via a simple, cross-platform supported, user-centric GUI. Overall, by simplifying and optimizing the biomarker candidate discovery process, MOAgent promises a harmonious blend of accuracy and accessibility, which will push the boundaries of interdisciplinary omic research.

## Supplementary Material



## Data Availability

All produced case study results in this paper, can be found and reproduced in and with the MOAgent virtual machine and the corresponding input data provided via Zenodo: https://zenodo.org/records/14446385

## Notes

Figure S1, example of feature expression table. Figure S2, example of a class annotation table. Figure S3, example of a patient annotation table. Figure S4, example of a UMAP output. Figure S5, example of a PCA output. Figure S6A and S6B, reliability evaluation using *P*-values (A) and SHAP-values (B). Figure S7, example of hierarchical clustered Kendall correlation heatmap. Figure S8, example of a Box plot visualizing the feature expression distribution across classes. (2) Supplementary Tables. Table S1, retrieved proteins with highest classification contribution of the MPN case study (PDF)
