# Supplementary material for: MultiOmicsAgent: Guided Extreme Gradient-Boosted Decision Trees-Based Approaches for Biomarker-Candidate Discovery in Multiomics Data
Source: J Proteome Res. 2025 May 26;24(6):2816–31. doi: 10.1021/acs.jproteome.4c01066 (PMC12150338; doi:10.1021/acs.jproteome.4c01066)
Supplement: Supplementary file 1 [file pr4c01066_si_001.pdf]

MultiOmicsAgent: Guided extreme gradient-boosted decision trees-based approaches for biomarker-candidate discovery in multi-omics data

Jens Settelmeier[1,2], Sandra Goetze[1-3], Julia Boshart[1], Jianbo Fu[1-3], Amanda Khoo[1], Sebastian N. Steiner[1], Martin Gesell[1], Jacqueline Hammer[1], Peter J. Schüffler[5], Diyora Salimova[6], Patrick G. A. Pedrioli[1-4,*] and Bernd Wollscheid[1-3,*].

[1]Institute of Translational Medicine at the Department of Health Sciences and Technology, ETH, 8093 Zurich, Switzerland

[2]Swiss Institute of Bioinformatics, 1015 Lausanne, Switzerland

[3]ETH PHRT Swiss Multi-Omics Center (SMOC), 8093 Zurich, Switzerland

[4]Department of Biology, ETH, 8093 Zurich, Switzerland

[5]Institute of Pathology, TUM School of Medicine and Health, Technical University of Munich, 81675 Munich, Germany

[6]Department for Applied Mathematics, Albert-Ludwigs-University of Freiburg, 79104 Freiburg, Germany

*To whom correspondence should be addressed: Patrick G. A. Pedrioli, E-mail: pedrioli@imsb.biol.ethz.ch, Phone: +41 44 633 21 95 and Prof. Bernd Wollscheid, E-mail: bernd.wollscheid@hest.ethz.ch, Phone: +41 44 633 36 84.

Supporting Information

1) Supplementary Figures for Visualizations
   - Figure S1, example of feature expression table
   - Figure S2, example of a class annotation table
   - Figure S3, example of a patient annotation table
   - Figure S4, example of a UMAP output
   - Figure S5, example of a PCA output
   - Figure S6A and S6B, reliability evaluation using P-values (A) and SHAP-values (B).
   - Figure S7, example of hierarchical clustered Kendall correlation heatmap
   - Figure S8, example of a Box plot visualizing the feature expression distribution across classes.

2) Supplementary Tables
   - Table S1, retrieved proteins with highest classification contribution of the MPN case study.

1. Supplementary Figures

Example input files

metabolite_expression_table.csv

	A	B	C	D	E	
1	files	Bin.9.98	Bin.9.94	Bin.9.90	Bin.9.86	Bi
2	P002	2.00E-05	2.00E-05	2.00E-05	2.00E-05	
3	P012	-3.00E-05	-3.00E-05	-3.00E-05	-1.00E-05	
4	P014	0.00024	0.00017	0.00016	0.00018	
5	P027	9.00E-05	0.00013	9.00E-05	9.00E-05	
6	P034	0.00012	0.00015	2.00E-05	3.00E-05	
7	P037	1.00E-05	-1.00E-05	2.00E-05	-3.00E-05	
8	P038	1.00E-05	1.00E-05	1.00E-05	-5.00E-05	
9	P041	-9.00E-05	-6.00E-05	-7.00E-05	1.00E-05	
10	P042	0.00015	8.00E-05	0.00017	3.00E-05	

Figure S1: Expression table, with the first column "files" corresponding to the sample file identifiers, the last column "class" corresponding to the sample class assignment, and all other columns named by the features corresponding to the feature expressions.

class_annotations.csv

	A	B
1	files	class
2	P002	patient
3	P012	patient
4	P014	patient
5	P027	patient
6	P034	patient
7	P037	patient
8	P038	patient
9	P041	patient
10	P042	patient

Figure S2: Class annotation table, with the first column “files” corresponding to the samples' file identifiers and the column “class” corresponding to their class assignments.

	A	B
1	files	PatientID
2	wildschm_B1909_024.mzML	14
3	wildschm_B1909_025.mzML	72
4	wildschm_B1909_026.mzML	100
5	wildschm_B1909_027.mzML	55
6	wildschm_B1909_029.mzML	7
7	wildschm_B1909_030.mzML	98
8	wildschm_B1909_032.mzML	15
9	wildschm_B1909_033.mzML	17
10	wildschm_B1909_036.mzML	108
11	wildschm_B1909_037.mzML	58
12	wildschm_B1909_039.mzML	94
13	wildschm_B1909_040.mzML	5
14	wildschm_B1909_042.mzML	76
15	wildschm_B1909_043.mzML	82
16	wildschm_B1909_044.mzML	81
17	wildschm_B1909_045.mzML	57
18	wildschm_B1909_046.mzML	104
19	wildschm_B1909_049.mzML	85
20	wildschm_B1909_050.mzML	99

Figure S3: (Optional) patient annotation table with the first column “files” corresponding to the file identifiers of the samples and the column “PatientID” to the individual the sample belongs to. This tabular file is used if several files (replicates) belong to the same individual (e.g. patient). Several files can be assigned the same PatientID, to let the algorithm know that the files are replicates of the same patient (individual).

Feature Analysis and Visualization

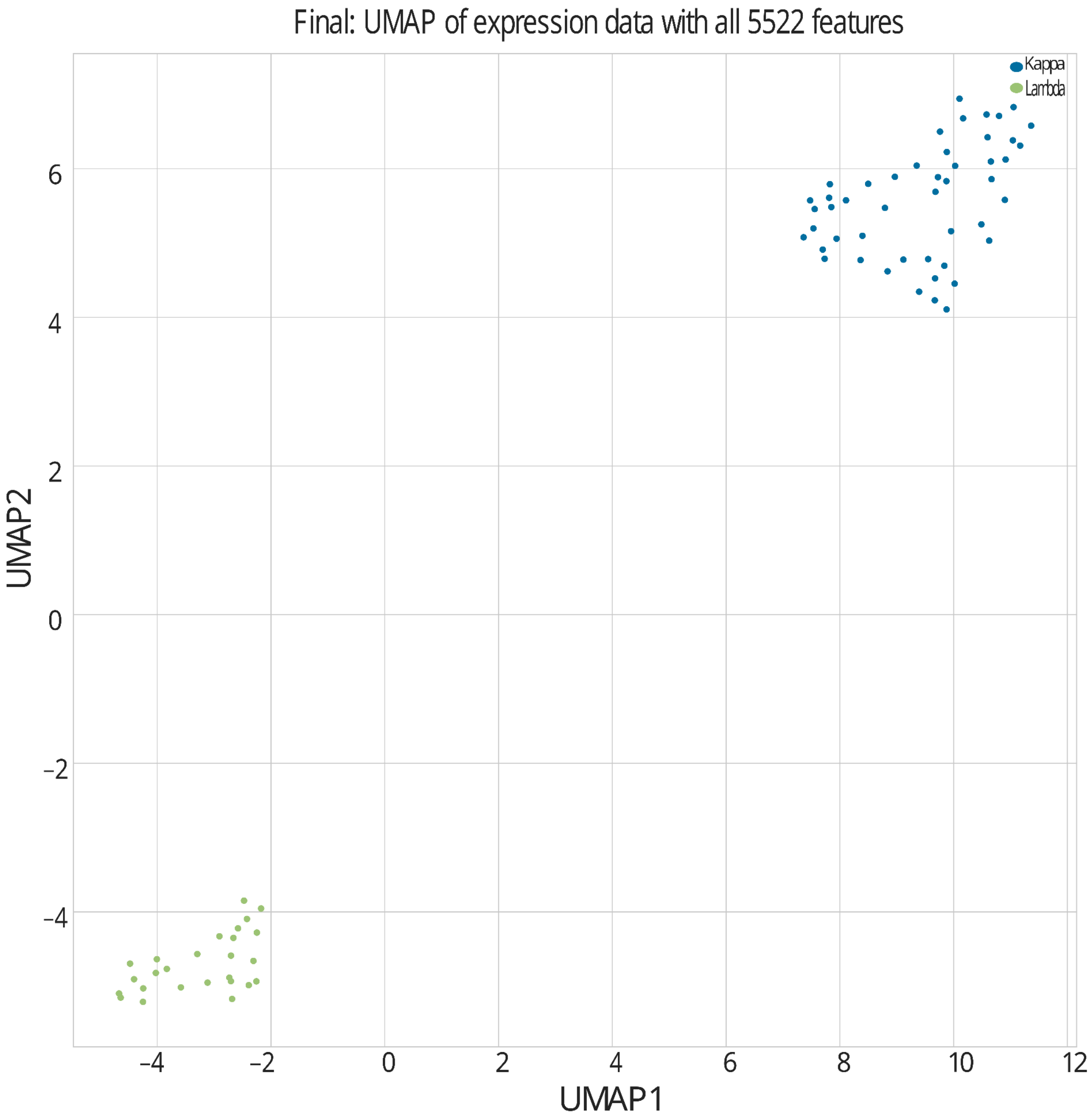

Figure S4: UMAP using all features of the Kappa-Lambda Protein case study.

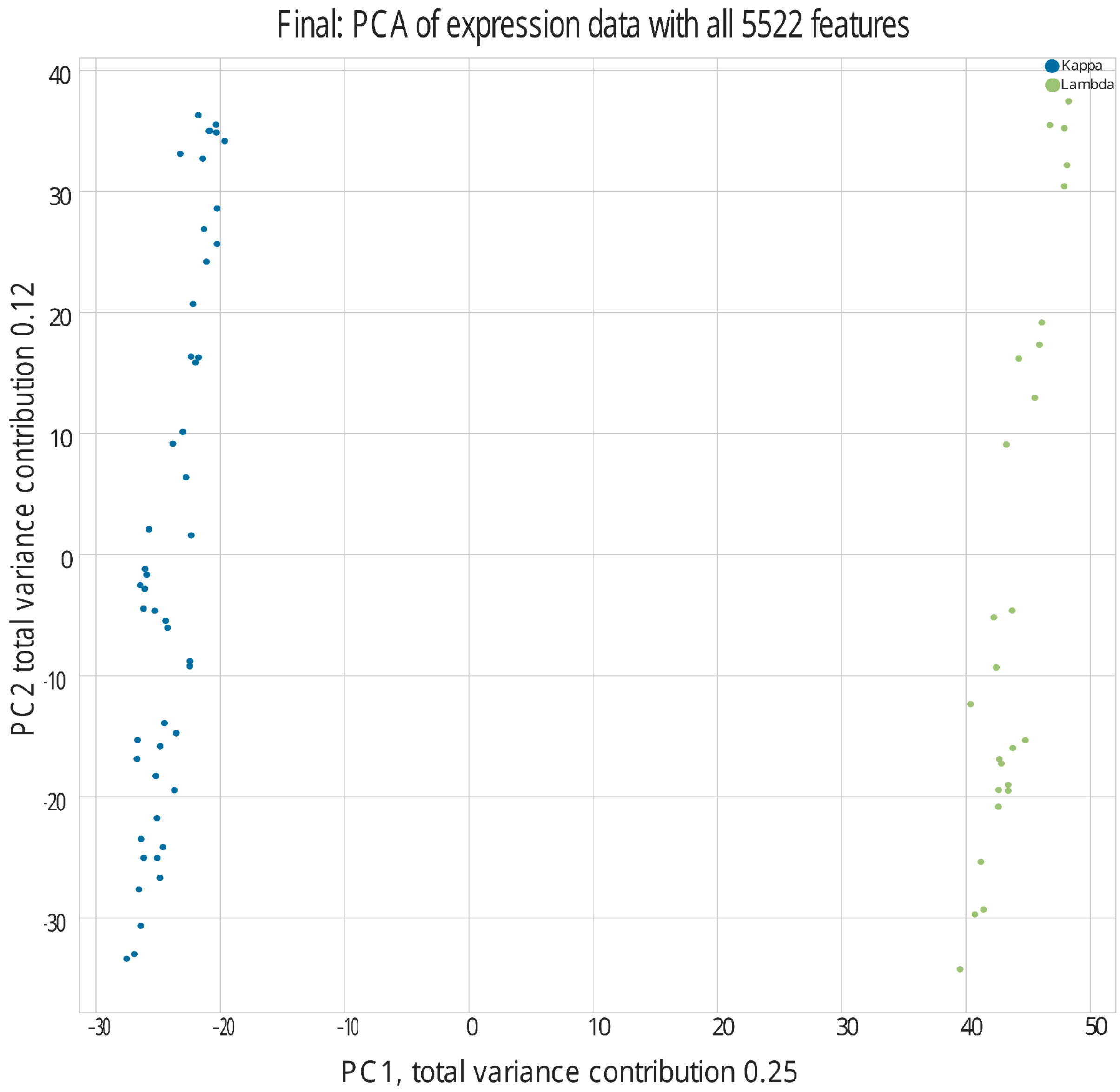

Figure S5: PCA using all features of the Kappa-Lambda Protein case study.

Feature Trustability Visualization

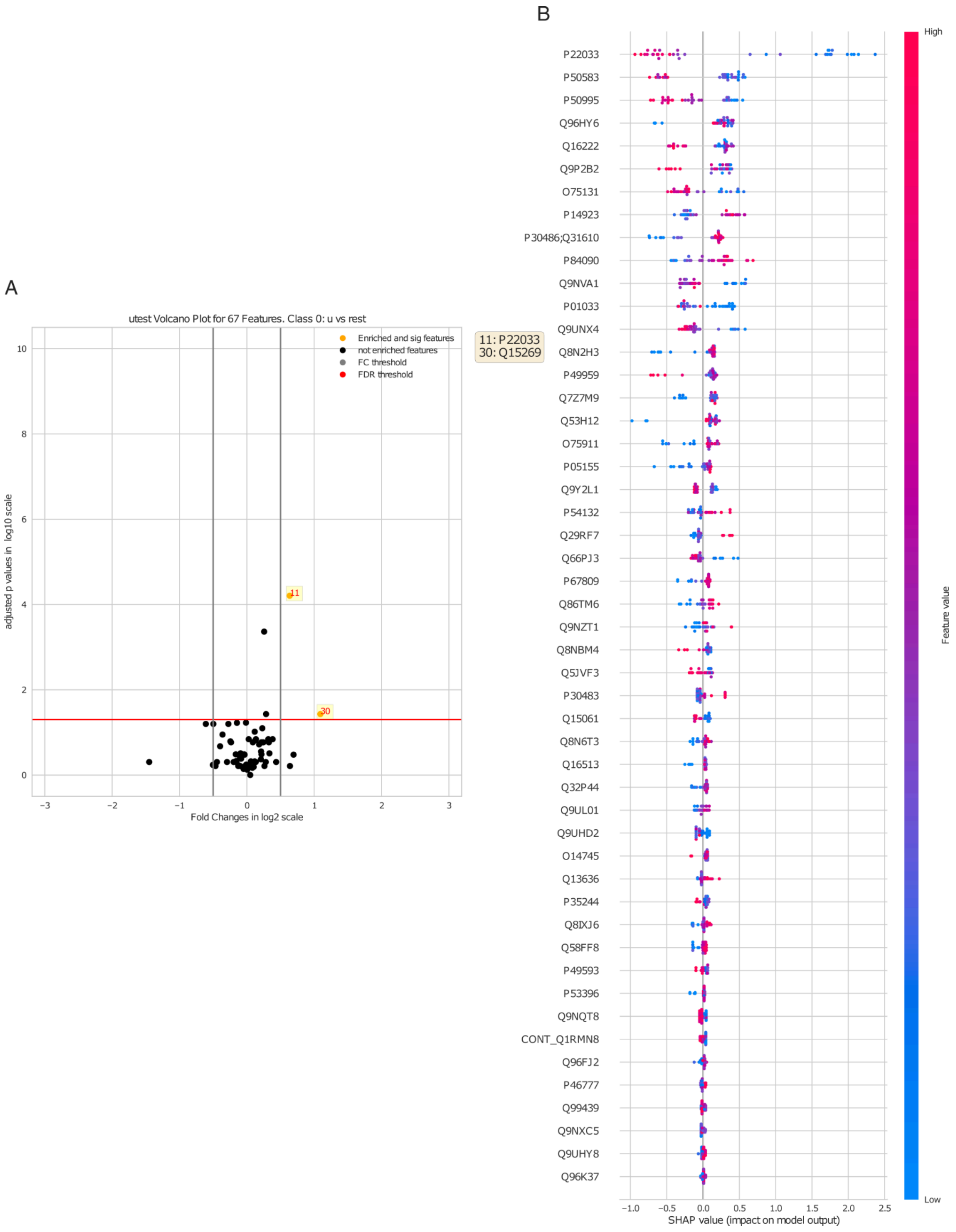

Figure S6: The volcano (S6A) and SHAP value (S6B) plots are examples of the output for the 67 MOAgent selected features in the MMA case study on the protein level.

Correlation Analysis and Feature Distribution:

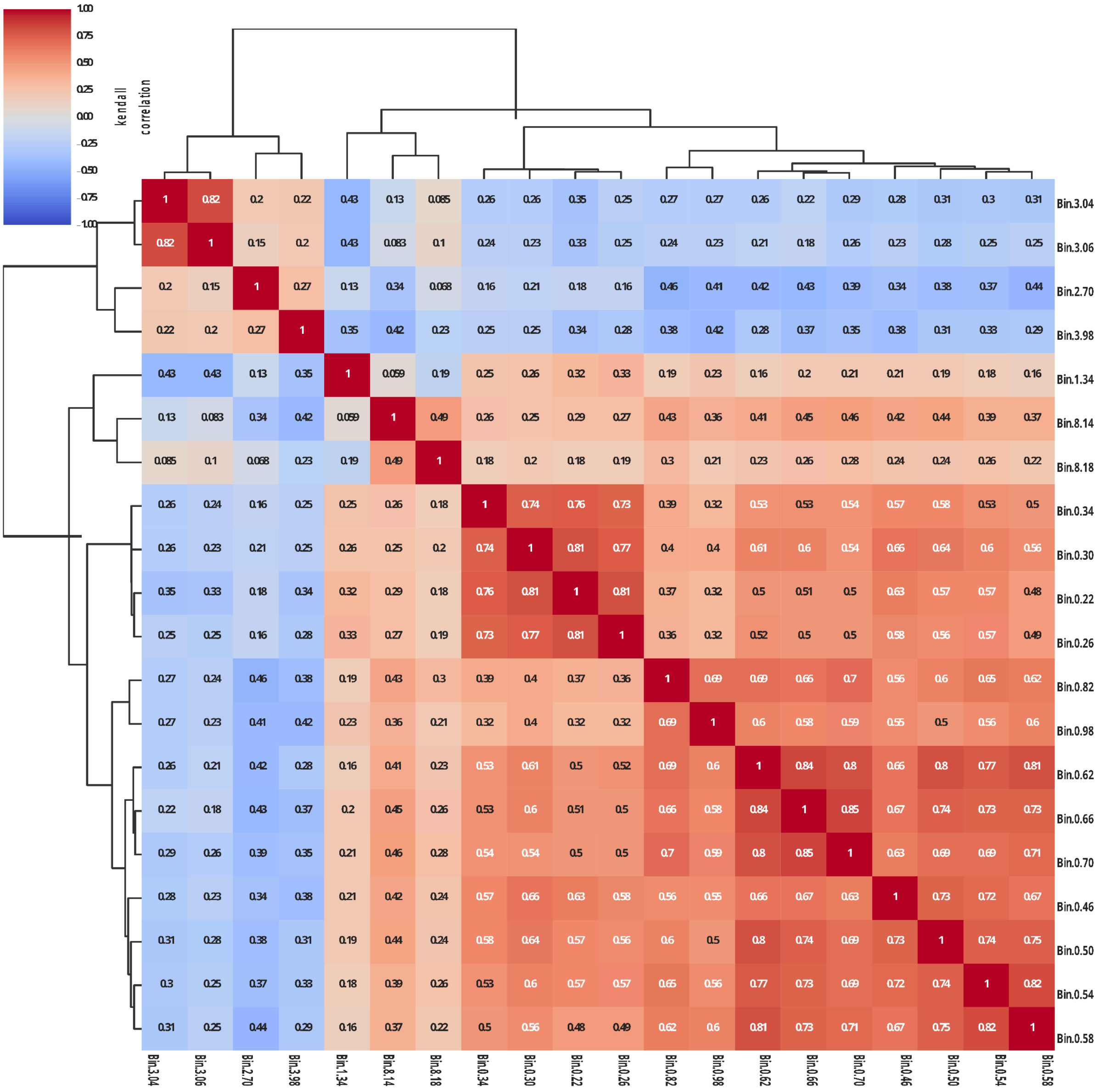

Figure S7: Example Kendall correlation heatmap and hierarchical clustering of the features selected by MOAgent in the GN cohort case study.

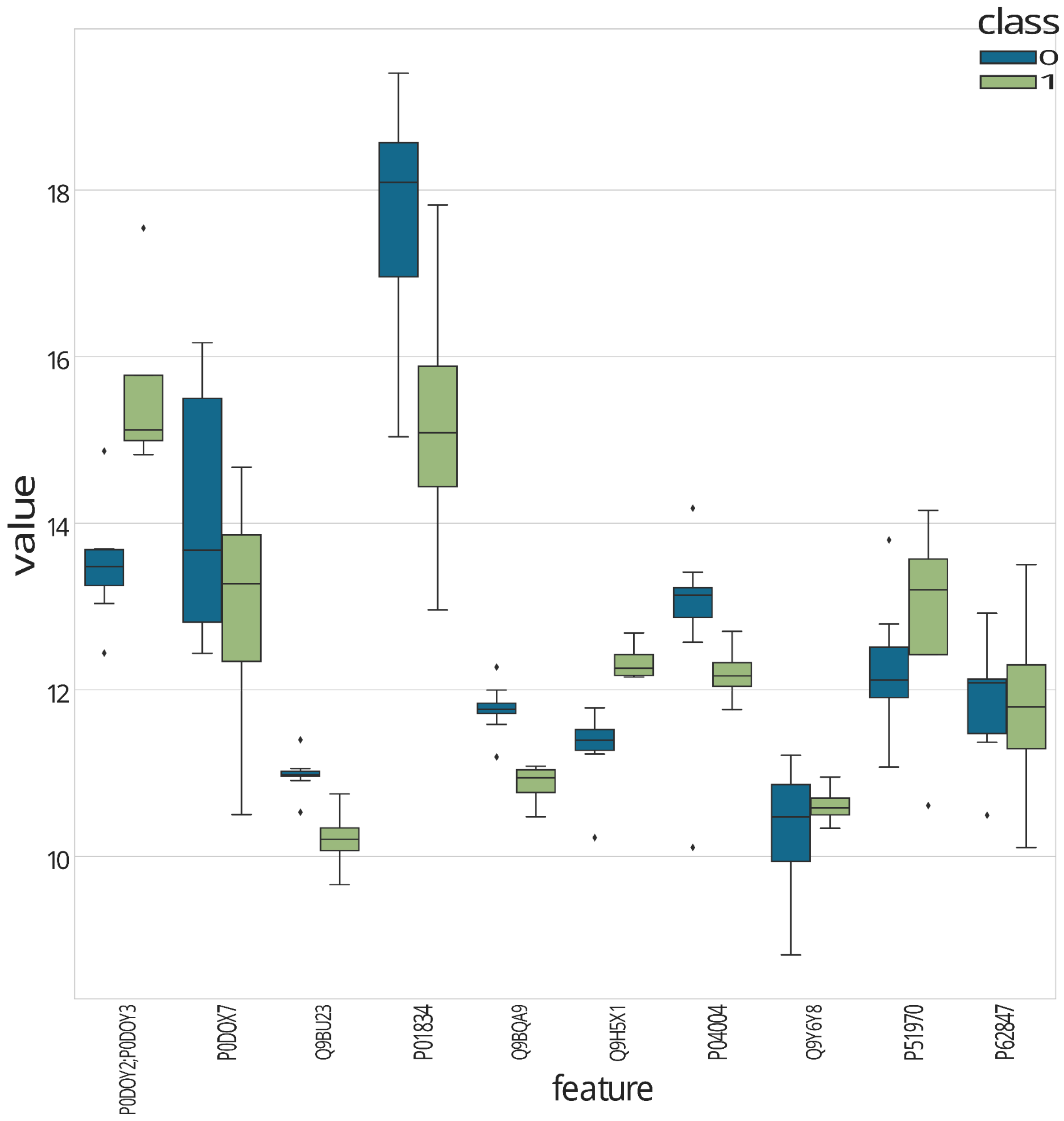

Figure S8: Box plot visualizations of the selected features by MOAgent in the Kappa-Lambda case study on protein level.

2. Supplementary Tables

ID from the original MPN study	Identified by Fragpipe	Selected by MOAgent (|correlation| > 0.5, adj. p < 0.01)
NEK5 (Q6P3R8)	no	no
MCM4 (P33991)	yes	no
ALPL (P05186)	yes	ALPL (P05186)
FH (P07954)	yes	TUFM (P49411)
CALR (P27797)	yes	CALR (P27797)
STYX (Q8WUJ0)	no	no
PSAT1 (Q9Y617)	yes	PSAT1 (Q9Y617)
ZNF735 (P0CB33)	no	no
COPS8 (Q99627)	yes	RFLNB (Q8N5W9)
CD59 (P13987)	yes	CD59 (P13987)
NAMPT (P43490)	yes	NAMPT (P43490)
ACSL1 (P33121)	yes	CKAP4 (Q07065)
S100A9 (P06702)	yes	no
MCM7 (P33993)	yes	KDM1A (O60341)
RFC2 (P35250)	yes	TAF6 (P49848)
POTEJ (P0CG39)	yes	no
PSTPIP1 (O43586)	yes	ATP6V1G1 (O75348)
PSMB8 (P28062)	yes	ALDH2 (P05091)

TPM3 (P06753)	yes	TPM3 (P06753)
STOML2 (Q9UJZ1)	yes	UQCRC2 (P22695)
CST7 (O76096)	yes	ALG1 (Q9BT22)

Table S1: The table provides an overview of the original 21 most phenotype discriminative proteins found in the original MPN study by Wildschutt et. al. and whether they were found by the protein identification and quantification platform Fragpipe. The last column provides from MOAgent identified most phenotype discriminative proteins which are correlated (using Kendall's Tau method) to the original proteins with an absolute correlation value exceeding 0.5 and significance filtered with Benjamini-Hochberg corrected p-values smaller than 0.01.
